# Peer review of "Impact of P fertilizer and arbuscular mycorrhizal fungi on forage legume growth, chlorophyll content and productivity"

_PeerJ, doi:10.7717/peerj.18955_

## Round 0.1 · original submission · Major Revisions

· Academic Editor

Major Revisions

Reviewers have covered all necessary topics. Make major revisions to your manuscript and resubmit.

·

Basic reporting

Overall, the English used in the manuscript is clear and of professional level. However, the authors need to revise the comments and inputs given under “Other inputs”. There is sufficient literature unless stated otherwise in comments under “other inputs”, the background and research problem are clear.
The structure of the manuscript is in line with scientific standards and tables are properly provided and referred to intext except for table 1. Raw data has been checked and meets the requirements, it does not appear to be corrupted or manipulated. Additionally, sufficient results cover the scope of the research questions.

Experimental design

The methodologies used in the research are in line with the research questions unless stated otherwise on “Other inputs”. The authors ensured scientific standards with their choice of methods. In this section, however, one or two details must be added to ensure repeatability.

Validity of the findings

The impact of the findings lies in the practical application for improving forage legume productivity, which is significant for smallholder farmers in South Africa. The novelty is evident in identifying the optimal phosphorus (P) fertilizer rate in combination with arbuscular mycorrhizal fungi (AMF) inoculation, and in demonstrating species-specific responses. This kind of study seems to be one of the first empirical attempts to establish legume pastures in communal arable lands of South Africa.

The study appears robust, with a well-designed split-split plot design and thorough statistical analysis using repeated measures ANOVA. Controls such as uninoculated plants and varying P fertilizer levels provide a clear comparative framework. The study, however, fails to discuss the limitations of the study, such as the greenhouse conditions versus field conditions, or any constraints in the scope of the study.

The conclusions are well-linked to the original research questions and the supporting results. They clearly state the influence of AMF inoculation and P fertilizer on legume growth, chlorophyll content, and productivity, with specific findings on the optimal P fertilizer rate and species-specific responses. However, there authors should consider removing the notion on policy making and rather stick with recommending future research scope.

Additional comments

Other Inputs
1. Line 19:
• Phosphorous deficiency limits forage productivity in South African soils
• Is this only a South African problem? If so show that on the manuscript title.
2. Line 21 to 24:
• Three legume species (Vigna unguiculata, Lablab purpereus and Mucuna pruriens) were grown under five P fertilizer levels (0, 20, 40, 60 and 80 kg P/ha) with or without AMF inoculation in the pots, resulting in 30 treatment combinations, each replicated 4 times.
• We are missing information telling us that this was a pot study and conducted in a controlled environment greenhouse.
3. Line 31:
• improved growth and productivity of
• “improved the growth and productivity”
4. Line 35 to 36:
• Remove “Plant height and stem diameter” and put “Legume forage productivity” Also, remove “P fertilizer rate” and put “Soil nutrient management”
5. Line 47:
• Australian government
• Put “the” in front of Australian government
6. Line 48 (and throughout the document):
• Do not personalise the writing.
7. Line 51:
• Phosphorus
• Needs to have a small letter ‘p” i.e. “phosphorus not Phosphorus”
8. Line 67:
• Phosphatase
• The P needs to be made in small letters.
9. Line 82 to 83:
• The study was conducted at the University of Fort Hare (32o 46' S 26o 50' E) greenhouse during January 2017.
• Rephrase to: A greenhouse pot experiment was conducted at the University of Fort Hare, Alice, Eastern Cape, South Africa (32o 46' S 26o 50' E) in January 2017.
10. Line 83 to 84:
• The experimental design was a split-split plot design (SSPD) with 3 x 2 x 5 factorial arrangement.
• Rephrase to: The experiment was laid out in a split-split plot design (SSPD) with a 3 x 2 x 5 factorial arrangement.
11. Lines 84 to 87:
• The SSPD comprised of three legume species (L. purpureus, M. purpureus and V. inguculata), two arbuscular mycorrhiza fungi levels (inoculated or uninoculated) and five P fertilizer rates (0, 20, 40, 60 and 80 kg P/ha), included as the main plot, subplot, and sub-sub plot, respectively.
• Rephrase: The main plot factors were three legume species while the subplot factors were arbuscular mycorrhiza fungi (inoculated and uninoculated) and the sub-sub plot factors were P fertilizer levels (0, 20, 40, 60 and 80 kg P/ha).
12. Line 86:
• (0, 20, 40, 60 and 80 kg P/ha).
• Why are the rates given in kg/ha but pots were used? Please represent it as the amount per pot.
13. Line 95:
• 20, 40, 60 or 80 kg/ha
• This is a replication of line 86.
14. Line 97
• Three seeds per pot were planted at 4-6 cm depth of soil and thinned to two after seed germination.
• Is this after germination/is it after emergence? Also, what was the space in between the seeds?
15. Line 99:
• Watering
• Replace with Irrigation.
16. Line 102:
• hand removal of any emerging weed.
• Replace with: Manual weeding.
17. Line 105:
• Base
• Which base? The base of the pot or the base of the plant?
18. Line 110:
• 30 of sowing by randomly selecting three leaves on each plant per pot
• The other growth measures do not tell us how many plants were selected and whether it was randomised when collecting the data.
19. Line 113:
• Bagged
• What type of bags were used?
20. Line 114:
• fresh weight of each forage component was determined
• With what instrument was this done?
21. Line 115:
• 65 oC for 48 hours and weighed to determine
• Are you sure that they were all dried at 48 hours? Dry weight is normally taken only at a constant weight, when repeated weighing eventually shows the same value.
22. Line 116 and throughout the document:
• leaf to stem ratio
• Use “leaf-to-stem ration”
23. Line 118:
• Firstly, data normality and homoscedasticity were assessed using Kolmogorov-Smirnof and Levenne’s tests
• Start the paragraph like: Normality and homoscedasticity of the data were assessed using Kolmogorov-Smirnof and Levenne’s tests
24. Line 127 and throughout the document:
• Overtime
• Replace with “over time”: Over time' generally refers to the passing of time within a given context. 'Overtime' is a specific concept related to working hours
25. Line 127 and throughout the document:
• ..since sowing..”
• This is misleading, the plant height and stem diameter were only observed after plant emergence.
26. Line 147:
• Again this is misleading, please see the above comment, there are no leaves at sowing.
27. Lines 144 to 145
• The AMF inoculated plants showed interspecific responses to P fertilizer over time.
• Please rephrase to be clear: Did the plant species respond differently to the treatments?
28. Line 145 and throughout the document:
• AMF inoculated
• Please ensure for all you write “AMF-inoculated”
29. Line 150:
• Low
• Replace with lower
30. Line 152:
• The results showed however, that the chlorophyll c
• Replace with: “However, the results also showed…´

31. Line 165:
• Remarkable
• Replace with “remarkably”
32. Line 177:
• Discussion
• The first part of the discussion needs to tell the reader if the soil properties, referring to table 1, were sufficient for the growth of the legumes.
33. Line 179 and throughout the document:
• Chlorophl
• Correct to chlorophyll
34. Line 187 to 188:
• Even at 0-20 kg P/ha, growth enhancement by AMF inoculation was minimal probably due to P deficiency.
• What is the mechanism of operation for this? Is it AMF that allows for P availability or is it enzymes produced by AMF that ensures this?
• If it is the latter please make it clear here, as enzyme activity can be said to be substrate-dependent whilst also species-dependent.
• Also, the study would have benefitted from an enzyme activity analysis
35. Line 190:
• This finding disagrees with several previous studies that suggest that AMF compensates for low soil P via enhancing acquisition of nutrients (e.g., Nouri et al. 2014).
• You indicated several studies; however, you gave 1 citation, please add a minimum of 3 citations.
36. Line 190:
• “we”
• Personalisation in science writing: “It can therefore be deduced that…..”
37. Line 194:
• interspecifically
• Rather change to “by species”
38. Line 196:
• Efficient
• Replace with efficiently
39. Line 200 to 202:
• Apart from enhancing P uptake, AMF enhances uptake of other essential nutrients (e.g., N, K and Fe) and reduces the uptake of salt ions, thereby stimulating vigorous plant growth and photosynthesis (Begum et al. 2019).
• This does not fit with the objectives of this study. The study is looking at phosphorous only.
40. Line 207:
• further depicted by higher leaf to stem ratio
• Replace to read: “further depicted by a higher leaf-to-stem”
41. Line 208 to 210:
• suggesting that the little available P was invested in leafiness rather than stem production. Generally, as legume plants grow, especially AMF inoculated plants with their tap root system, have a better access to nutrients on the deeper zones of the soil profile.
• Plants often allocate more resources to their roots rather than their leaves when phosphorus (P) is limited in the soil. Root data would have assisted in explaining these responses.
42. Line 209 to 210
• Generally, as legume plants grow, especially AMF inoculated plants with their tap root system, have a better access to nutrients on the deeper zones of the soil profile
• You did not provide data on root growth, please put a citation to this as it is not arising from this research. Although it is true, hence also the above comment.
43. Line 212 and 213:
• stunt legume growth, but also inhibits photosynthesis.
• Is there a relationship or assumed relationship between these two?
44. Line 231:
• Our results show, however,
• Swap: “However, the results show”. Do not personalise academic writing.
45. Line 237 to 239:
• These findings are a basis for soil nutrition management of forage legume pastures and may play a crucial role in policy making with regards to pasture establishment in communal arable lands of South Africa.
• The link to policymaking is unclear, please revise and keep the potential contributions purely scientific and practice based. Also, provide scope for future research.

·

Basic reporting

The manuscript entitled “Impact of Phosphorus fertilizer and arbuscular mycorrhizal fungi on forage legume growth, chlorophyll content and productivity” provides an overview of research investigating the impact of arbuscular mycorrhizal fungi (AMF) on three forage species: Vigna unguiculata, Lablab purpureus, and Mucuna pruriens, grown under five levels of phosphorus application. The study's key findings suggest that AMF application is beneficial for improving phosphorus (P) availability to plants. The authors hypothesize this by determining chlorophyll content, fresh and dry weights of plants, and their leaf-to-shoot ratio. However, several critical aspects of the manuscript need improvement.
Abstract: Please include key values in the abstract and results sections to clearly delineate the exact contributions and potential applications of the study. This will provide a more precise and comprehensive understanding of the findings.
Keywords: Revise keywords and add more specfic and novel keywords with broader meanings (5-7)
Scientific Background and Literature Review:
The scientific background is insufficiently explained. The manuscript should provide a more comprehensive overview of previous research on AMF and phosphorus interactions in forage legumes. Many other researchers have explored this idea using more advanced techniques with different forage crops. It is essential to review this literature carefully to identify actual research gaps and justify the novelty of your study. Ensure that the introduction contextualizes your research within the broader field and clearly states the specific questions or hypotheses your study aims to address.
Materials and Methods:
The materials and methods section lacks the necessary detail to ensure reproducibility. Provide comprehensive descriptions of the experimental setup. Detail the time of day for chlorophyll measurements. Please address how the success of AMF inoculation was verified and also include root growth parameters and mycorrhizal infection rates in the roots of the three forage crops. These parameters are crucial for confirming successful mycorrhizal establishment.
Results:
The results section should include key values to clearly demonstrate the impact of AMF on phosphorus solubilization and availability to each plant species. Present root growth parameters and mycorrhizal infection rates to substantiate the claim of successful AMF establishment. This data is essential for validating the effectiveness of the AMF treatment.
Discussion:
Strengthen the discussion by integrating your findings with the latest references. Provide a thorough explanation of how your results compare with previous studies and what new insights they offer. Address any limitations of your study, such as environmental conditions, potential confounding factors, or methodological constraints. Discuss how these limitations might affect the interpretation of your results. Highlight future research directions and practical applications of your findings.
Figures and Supplementary Material:
The manuscript currently lacks figures. Adding visual representations of your data, such as graphs and charts, will help convey your findings more effectively. Please include pictorial glimpses of the research work in the supplementary material. This could include images of the experimental setup, AMF inoculation process, and plant growth stages.
Conclusion:
Please add future recommendations in the conclusion section.
References:
Please double-check the references to ensure that their style is consistent and homogeneous throughout the manuscript.
Due to these significant issues, the quality of the manuscript currently does not meet the standards of PeerJ and requires major revisions.
Specific comments
1)Explain how this statement in Line No. 49-50 is justified while it was a pot experiment “To the best of our knowledge, this was the first empirical attempt to establish legume pastures in communal arable lands of South Africa.
2)Please mention the references in the Line No. 51 as there are studies reporting the influence of AFM on phosphorus availability to forage plants “Hence, little has been done to ascertain factors that limit legume establishment and productivity.”
3)Line 73-74 please clarify the statement in more realistic terms“This deprives us an opportunity to design appropriate soil nutrition management program for sustainable legume pasture establishment and production.
4)Line No. 82-83 Please clarify that a study that was conducted seven years back how fits under current climatic conditions. “The study was conducted at the University of Fort Hare (32o 46’ S 26o 50’ E) greenhouse during January 2017.
5)Line 84 How the authors will justify it for a pot experiment? “The experimental design was a split-split plot design (SSPD) with 3 x 2 x 5 factorial arrangement.”
6)Justify it please in Line No. 86-87 “included as the main plot, subplot, and sub-sub plot, respectively.”
7)Line No. 100 please explain the photoperiod under green house conditions supplied to plants and sowing dates with months and year “Watering was done once a day in the morning to maintain moisture at 50% field capacity to avoid leaching.”
8)In lines 204-206, the authors state, "This could imply that despite low P content at 0 and 20 kg P/ha, as plants grew, they were able to acquire more P and efficiently channel it to photosynthetic rather than non-photosynthetic apparatus." How do the authors justify this statement without directly measuring the photosynthetic rate?
9)In lines 220-221, the authors present a controversial statement: "However, the leaf:stem ratio was highest for uninnoculated relative inoculated plants (Table 4), suggesting that the latter invested more P not only to leaf production but also to stem production." If the leaf surface area is higher in uninnoculated plants, what is the role of AMF in this case? This statement needs further clarification.
10)Please include detailed information in the methodology section regarding the soil parameters listed in Table 1, including the specific protocols used to determine them.
11)Please also provide the raw data of leaf dry matter and stem dry matter as well as soil analysis in supplementary materials.

Experimental design

The experimental design of the manuscript requires clarification to ensure consistency and coherence. The current description contains conflicting statements about the design and setup, leading to confusion. To address these issues, the following points should be elaborated and expanded, In line 83, the authors mention the experiment was conducted using a split-split plot design. However, in line 89, they describe it as a pot trial, and in line 98, they mention that the pots were arranged randomly. These statements appear contradictory and need to be reconciled to provide a clear and consistent description of the experimental design.
Please clearly define whether the study was conducted using a field trial with a split-split plot design, a pot experiment, or a combination of both. Provide a rationale for the chosen design and explain how it was implemented in practice. Please check the manuscript for consistency and coherence in describing the experimental design. Ensure that all sections of the manuscript (introduction, materials and methods, results, discussion) align with the clarified experimental setup.
The statistical analysis appears to be appropriate; however, to establish a comprehensive relationship between the different forage crops, phosphorus application rates, and inoculated versus non-inoculated plants, the authors are strongly encouraged to include a correlation plot.

Validity of the findings

The findings are promising. However, there is a lack of information regarding the influence of mycorrhizal fungi on phosphorus availability in the soil and its absorption by plants. It is unclear how the authors assumed that AMF established in the roots of the plants without providing solid evidence. Therefore, the authors are advised to include this crucial parameter in the revised version of the manuscript. Additionally, the authors should investigate and report on the influence of AMF on root growth and rooting depth.

Additional comments

The manuscript can be accepted after major revisions.

·

Basic reporting

Dear author/authors;
The study conducted is suitable for publication, original, and interesting. However, there are some deficiencies, both generally and specifically in the manuscript sections, as noted below. Addressing these deficiencies will make the study publishable. In the introduction section of the manuscript, sentences have been used that imply the study is local. The study was conducted to contribute to solving a global problem, not just a local one. These sentences in the abstract and introduction should be replaced with general statements. Another deficiency is the lack of tables/graphs showing the effects of mycorrhiza and phosphorus applications individually and in combination. Adding these and discussing them in the context of the literature will make the manuscript clearer and more comprehensive.
Best regards.

Abstract
Phosphorus deficiency limits productivity not only in South African soils but in all soils in the world. Therefore, global rather than local expressions should be used. The study conducted and the results obtained appear to be universal rather than local. I recommend using appropriate sentences reflecting this in the abstract and introduction sections.
Keywords
The words used in the title should not be used in the keywords. Additionally, the keywords should be listed in alphabetical order.
Introduction
The introduction section of the manuscript is generally well written. The cited sources should be original research articles rather than reviews. Some sentences used are similar to the sentences cited by the referenced sources. As previously mentioned, the writing should be in a global context. Since the study is not specific to South Africa, you should discuss the global situation of the issue, not just the situation in your country. Additionally, providing information on the effects of mycorrhiza and phosphorus or microorganisms and nutrients on time-dependent changes in plant height, stem diameter, and chlorophyll observed in the study, if available in the literature, will enhance the quality of the manuscript.
Lines 41-53: When discussing animal feed production and phosphorus deficiency in soils, which is a problem not only for South Africa but for the entire world, global statements should be used.
Line 48: I think it should be “Davis et al.”, not “Davies et al.”

Experimental design

Materials & Methods
The Materials and Methods section is well-written, but there are some deficiencies. Addressing these deficiencies will make the manuscript publishable. Additionally, as understood from Lines 84-89, only one pot was used in each replicate. This reduces the statistical reliability of the study to some extent. However, the cultivation of 2 plants per pot partially mitigates this deficiency.
Line 94: The proportions of the species included in the mycorrhiza mixture used should be specified.
Line 94: The method of applying mycorrhizae to the seeds and/or soil should be explained in more detail.
Line 95: The method of calculating the fertilizer amount used per hectare for each pot should be clearly specified. Was it based on volume, surface area, or another method? Please provide detailed information.
Line 96: Is the depth of the pot and the soil inside the pot correctly stated? If not, it should be corrected. If it is correct, an explanation should be provided on how the seeds were sown at a depth of 4-6 cm as mentioned in Line 97 and at what depth the plant roots grew.
Line 103: Information about other fertilizers given to the plants should be provided.

Validity of the findings

Results and Discussion
The results are well-written, clear, and sufficient according to the tables in the manuscript. However, tables examining the individual and dual combination effects of cultivar, mycorrhiza, and phosphorus fertilizer (cultivar x mycorrhiza, cultivar x phosphorus, mycorrhiza x phosphorus) are missing. These tables need to be added. This will allow for the observation of the individual, dual, and triple effects of the applications.
Lines 186-187: The information in the mentioned lines was not produced in the referenced study. The study cited as "Tobisa & Uchida, 2017" similarly references other studies in a similar sentence. Similarly, citations should be made to the original studies where the work was conducted.
Lines 188-190: In the relevant lines, the phrase "several previous studies" is used, but only one study is cited. It is unclear which studies are being referred to. References to the relevant studies should be provided at the end of the sentence.
Lines 190-192: In your study, mycorrhiza was found to be more effective at a phosphorus dose of 40 kg P/ha. This finding needs to be discussed more effectively with the literature.
Lines 203-221: The brave claims in the relevant lines need to be discussed with the literature.
Lines 221-224: If sentences are taken from the same study, it is sufficient to cite it once at the end of the last sentence.

---

## Round 0.2 · Minor Revisions

· Academic Editor

Minor Revisions

There are still issues in your work that need correction. In addition to the reviewers' comments, please consider the following: Line 259: Mitran et al. (2028), are you referring to a future study? Please correct the date.

·

Basic reporting

The authors need not convert the amount of phosphorous applied per pot to phosphorous. If they insist on converting to per Ha then they should use the figures of 2 million to 2.2 million kg of soil in the top 15 cm of a hectare, to do their calculations. Using the surface area in a pot is not advisable, hence scientific writing does not permit this conversion.

Due to this, the entire discussion of the manuscript needs to be re-written to factor in the values obtained from this new calculation. or use the per-pot values and compare the findings with other pot studies.

Experimental design

The design is solid, it is just the fertilizer applications which need to be revised.

Validity of the findings

The findings will be valid provided that the aforementioned calculation is done.

Additional comments

See on attached manuscript

·

Basic reporting

I am pleased to see that the authors have successfully implemented all the recommended revisions, with the exception of one key suggestions. The methodologies employed to measure the parameters presented in the table should be included. In addition, a detailed description of the soil results in Table 1 should be provided, with appropriate citation and reference to the table within the text.
Moreover, authors are suggested to add the full forms of all abbreviations used in Figures 1 and 2 should be added as footnotes to the respective figures for clarity and technical accuracy.
It is recommended that the authors conduct a thorough review of the manuscript to identify and correct any typographical and grammatical errors. The article can be considered for publication once these suggestions have been incorporated.

Experimental design

The experimental design appears to be promising and technically sound.

Validity of the findings

The findings presented in the study are scientifically valid and supported by appropriate methodologies, ensuring that the conclusions drawn are credible and reliable.

·

Basic reporting

Dear author/authors;
The study conducted is suitable for publication, original, and interesting. However, there were some deficiencies, both generally and specifically in the manuscript sections, but many of them has been resolved. However, there are some deficiencies listed below. Once these deficiencies are addressed, the study will be suitable for publication. In addition, no space was left between some words and at the end of some sentences after the “point”. The text should be examined in this respect. Finally, two different methods have been chosen for the use of commas throughout the text. For example; (Apple, pear, orange, and banana) and (Apple, pear, orange and banana). One of these methods should be chosen and applied throughout the text.
Best regards.
Abstract
Lines 31-33: The sentence seems complicated. There are similar sentences throughout the Manuscript. Such sentences need to be converted into a more understandable form.
Lines 33-35: It should be explained which applications and which measurement the values specified in the sentence in question were obtained from. Such as data obtained on the 30th, 60th or 90th day.
Keywords
Introduction
There is a lot of self-citation in the section. I think there are many research articles that related to manuscript’s topic. Please change some of them with other ones, especially international ones.
Line 59: Please convert “Mpongwana et al. 2023ab” to “Mpongwana et al. 2023 a,b” or “a;b”.
Line 71: Is “Mpongwana et al. 2023 “a” or “b”?

Experimental design

Materials & Methods
Line 133: Is “Mpongwana et al. 2023 “a” or “b”?

Validity of the findings

Results
There are a lot of “AMF-AMF inoculated” term in the section. What is that mean?
Lines 231-233: Are there any shortcomings? The sentence has 4 features but 3 "r" values.
Discussion
Lines 235-283: The paragraph is too long.
Lines 291-294: The same publication should not be cited twice in consecutive sentences. A single citation at the end of the second sentence is sufficient.
Conclusion
Line 334: The species name is used in its full form "Mucuna pruriens" when first used. In subsequent uses, the abbreviated form "M. pruriens" should be used. If similar situations occur, they should be corrected.

Additional comments

References
Some citations are not included in the references section.

---

## Round 0.3 · Minor Revisions

· Academic Editor

Minor Revisions

Your manuscript still needs some final corrections

·

Basic reporting

This is a good topic; however, authors keep making mistakes with their treatments and the units that they used which are very important in the science community.

I suggest that all the authors reread the paper and fix these mistakes along with the English mistakes that make the paper difficult to follow.

Experimental design

The experimental design is of good scientific standards and the methodologies are well written.

Validity of the findings

The findings seem valid enough for publication, they will form the basis for more research in AMF inoculation.

Additional comments

No

·

Basic reporting

The authors have made commendable efforts to address all the queries and concerns raised during the second round of revisions. The revisions have substantially improved the quality and clarity of the manuscript, ensuring it meets the journal's standards for publication.

Experimental design

The experimental setup is appropriate and adequately supports the research objectives.

Validity of the findings

Findings seems valid

·

Basic reporting

The researchers have made the corrections indicated in the manuscript. However, there are some spelling and layout errors listed below. Once these errors are corrected, the manuscript can be published.

1) There are no spaces between words or abbreviations in the line numbers specified below. The lines should be examined and errors should be corrected. In addition, similar errors not specified here should be corrected.

Lines: 58, 98, 105, 167, 202, 233, 235, 242, 247, 248, 253, 260, 269, 283, 286, 325, and 332.

2) Line 103: The signs ")" and "." are not used at the end of the sentence ending with the word (Table).

3) Line 137: Parentheses are used twice.

4) In the text that will be the decimal separator, comma "," and point "." are used together. One of them must be selected and used throughout the text.

5) Line 116: Not "mycorrhiza". You should use "mycorrhizal"

6) Lines 122-123: There is a incorrect sentence.

7) Line 184: There are some sentences should be delete.

8) Lines 228, 252: I think the tittles are mixed up.

Experimental design

There are no noticeable shortcomings left in the experimental design.

Validity of the findings

There is no problem with the findings.

Additional comments

The article can be published by correcting the simple deficiencies and errors mentioned above.

---

## Round 0.4 · accepted · Accept

· Academic Editor

Accept

Your manuscript has been accepted after your final revisions.